# Enrichment Reveals Extensive Integration of Hepatitis B Virus DNA in Hepatitis Delta Virus-Infected Patients

**DOI:** 10.1093/infdis/jiae045

**Published:** 2024-01-25

**Authors:** Johan Ringlander, Lucia Gonzales Strömberg, Joakim B Stenbäck, Maria E Andersson, Sanna Abrahamsson, Catarina Skoglund, Maria Castedal, Simon B Larsson, Gustaf E Rydell, Magnus Lindh

**Affiliations:** Department of Infectious Diseases, Institute of Biomedicine, Sahlgrenska Academy, University of Gothenburg, Gothenburg, Sweden; Department of Infectious Diseases, Institute of Biomedicine, Sahlgrenska Academy, University of Gothenburg, Gothenburg, Sweden; Department of Infectious Diseases, Institute of Biomedicine, Sahlgrenska Academy, University of Gothenburg, Gothenburg, Sweden; Department of Infectious Diseases, Institute of Biomedicine, Sahlgrenska Academy, University of Gothenburg, Gothenburg, Sweden; Bioinformatics and Data Centre, Sahlgrenska Academy, University of Gothenburg, Gothenburg, Sweden; The Transplant Institute, Sahlgrenska University Hospital, Gothenburg, Sweden; Sahlgrenska Academy, Institute of Clinical Sciences, University of Gothenburg, Gothenburg, Sweden; The Transplant Institute, Sahlgrenska University Hospital, Gothenburg, Sweden; Sahlgrenska Academy, Institute of Clinical Sciences, University of Gothenburg, Gothenburg, Sweden; Department of Infectious Diseases, Institute of Biomedicine, Sahlgrenska Academy, University of Gothenburg, Gothenburg, Sweden; Department of Infectious Diseases, Institute of Biomedicine, Sahlgrenska Academy, University of Gothenburg, Gothenburg, Sweden; Department of Infectious Diseases, Institute of Biomedicine, Sahlgrenska Academy, University of Gothenburg, Gothenburg, Sweden

**Keywords:** hepatitis B virus, HBV integrations, hepatitis delta virus, deep sequencing, HBV RNA enrichment, HBsAg

## Abstract

**Background:**

Hepatitis B virus (HBV) DNA may become integrated into the human genome of infected human hepatocytes. Expression of integrations can produce the surface antigen (HBsAg) that is required for synthesis of hepatitis D virus (HDV) particles and the abundant subviral particles in the blood of HBV- and HDV-infected subjects. Knowledge about the extent and variation of HBV integrations and impact on chronic HDV is still limited.

**Methods:**

We investigated 50 pieces of liver explant tissue from 5 patients with hepatitis D-induced cirrhosis, using a deep-sequencing strategy targeting HBV RNA.

**Results:**

We found that integrations were abundant and highly expressed, with large variation in the number of integration-derived (HBV/human chimeric) reads, both between and within patients. The median number of unique integrations for each patient correlated with serum levels of HBsAg. However, most of the HBV reads represented a few predominant integrations.

**Conclusions:**

The results suggest that HBV DNA integrates in a large proportion of hepatocytes, and that the HBsAg output from these integrations vary >100-fold depending on clone size and expression rate. A small proportion of the integrations seems to determine the serum levels of HBsAg and HDV RNA in HBV/HDV coinfected patients with liver cirrhosis.

## Teaser

Explant tissue deep RNA sequencing showed that HBV DNA integrates extensively, but great variation in S-RNA levels suggests that a small part of the integrations determines serum levels of HBsAg and HDV RNA in HBV/HDV coinfected patients with liver cirrhosis.

Chronic infection with hepatitis B virus (HBV) is a major cause of liver disease, including hepatocellular carcinoma (HCC) [1]. The viral replication originates from an episomal genome (covalently closed circular DNA [cccDNA]) in the nucleus of infected cells [2]. A linear form of the HBV genome may become integrated into chromosomal DNA of infected hepatocytes [3–6]. The integrated genome cannot be transcribed to full-length pregenomic RNA and therefore does not allow replication of the virus, but transcription can occur of viral RNA species that code for the large, middle-sized, and small surface antigens (L-HBsAg, M-HBsAg, and S-HBsAg) and the X protein [7–10].

Hepatitis D virus (HDV) is an important cause of liver cirrhosis and HCC. HDV has a small circular RNA genome and requires HBsAg to form infectious virus particles. It does, however, not seem to require replicating HBV [11] because HBV integrations alone may be a source for HBsAg [4, 5, 12]. Studies have also shown HBsAg-independent HDV RNA cell-to-cell spread [13, 14] and there are delta-like viruses that replicate in the absence of HBsAg [15]. However, recent work shows that HBsAg clearly matters in HDV infection as the recently introduced treatment, the entry inhibitor bulevirtide that blocks HBsAg from binding the viral receptor sodium taurocholate co-transporting polypeptide (NTCP), reduces HDV RNA levels [16].

Previous studies of both HBV monoinfection and HBV/HDV coinfection have shown that HBV integrations are frequent and highly expressed, but published findings may still be underestimations of viral DNA and RNA [4, 8], because the high amount of human nucleic acids in liver tissue consumes a large proportion of the deep-sequencing capacity.

The aim of the present study was to further investigate the expression of chimeric human/HBV RNA in HDV coinfection, and study how this might correlate with serum levels of HDV RNA and HBsAg. To improve the identification of HBV RNA from integrations we developed and applied a novel strategy for the Ion Torrent platform, which uses polymerase chain reaction (PCR) to enrich RNA containing HBV sequences upstream of the typical junction point [8]. We combined this short-read deep sequencing with digital droplet PCR (ddPCR) to measure tissue levels of HDV RNA and with Nanopore sequencing to obtain long reads from HBV transcripts.

## METHODS

### Patients and Samples

The patients in this study (n = 5) had received a liver transplant because of HDV-induced liver disease. One of them (patient 3) earlier had very high serum levels of HDV RNA (> 7 log_10_ copies/mL) but was HDV RNA negative in serum at the time of transplantation. They all had liver cirrhosis and one had HCC. Ten pieces from each explanted liver were used for Ion Torrent sequencing and ddPCR. Patient characteristics are presented in Table 1. Three samples were used for Nanopore sequencing.

**Table 1.** Characteristics of the Patients

Characteristics	Patient 1	Patient 2	Patient 3	Patient 4	Patient 5
Indication for transplantation	Cirrhosis	Cirrhosis, HCC	Cirrhosis, acute on chronic hepatitis	Cirrhosis	Cirrhosis
Age, y	34.9	47.2	54.5	53.3	66.2
Sex	Male	Male	Male	Male	Male
Geographic origin	East Europe	Middle East	West Africa	Middle East	Middle East
HBV genotype	D	D	E	D	D
Serum viral levels					
Log HBV DNA IU/mL^a^	Approximately 1.0^b^	Undetected	1.05	Undetected	Undetected
Log HBsAg IU/mL^c^	3.91	2.70	3.09	3.07	3.43
Log HDV RNA copies/mL	6.53	3.94	Undetected	4.99	6.02
Antivirals prior to transplantation	…	Entecavir 2 mo	…	Tenofovir 2 y	…
Number of pieces analyzed	10	10	10	10	10

Abbreviations: HBsAg, hepatitis B surface antigen; HBV, hepatitis B virus; HCC, hepatocellular carcinoma; HDV, hepatitis D virus.

^a^By Cobas Taqman, Roche.

^b^Detected, but below quantification range.

^c^By Abbott Architect.

### Serum Markers

HBV DNA levels were measured by Cobas 6800 (Roche Diagnostics), HBsAg levels by Alinity (Abbott), and HDV RNA levels by real-time PCR as previously described [17].

### Nucleic Acid Extraction

Prior to analyses of RNA in liver tissue by Ion Torrent sequencing or ddPCR we used the RNeasy mini kit (Qiagen) for nucleic acid extraction according to manufacturer's instructions. Prior to real-time PCR quantification of HBV S DNA, HBV S RNA, and β-globin DNA, we performed total nucleic acid extraction with a Magnapure instrument (Roche). Tissue homogenization was performed using a Magnalyzer (Roche).

### Digital Droplet PCR and Real-Time PCR in Liver Tissue

Quantification of HDV RNA in liver tissue was performed with ddPCR (Bio-Rad), and quantification of HBV S DNA and RNA by real-time PCR, as previously described [9, 17].

### PCR Enrichment of HBV RNA and Ion Torrent Sequencing

**Figure 1.**
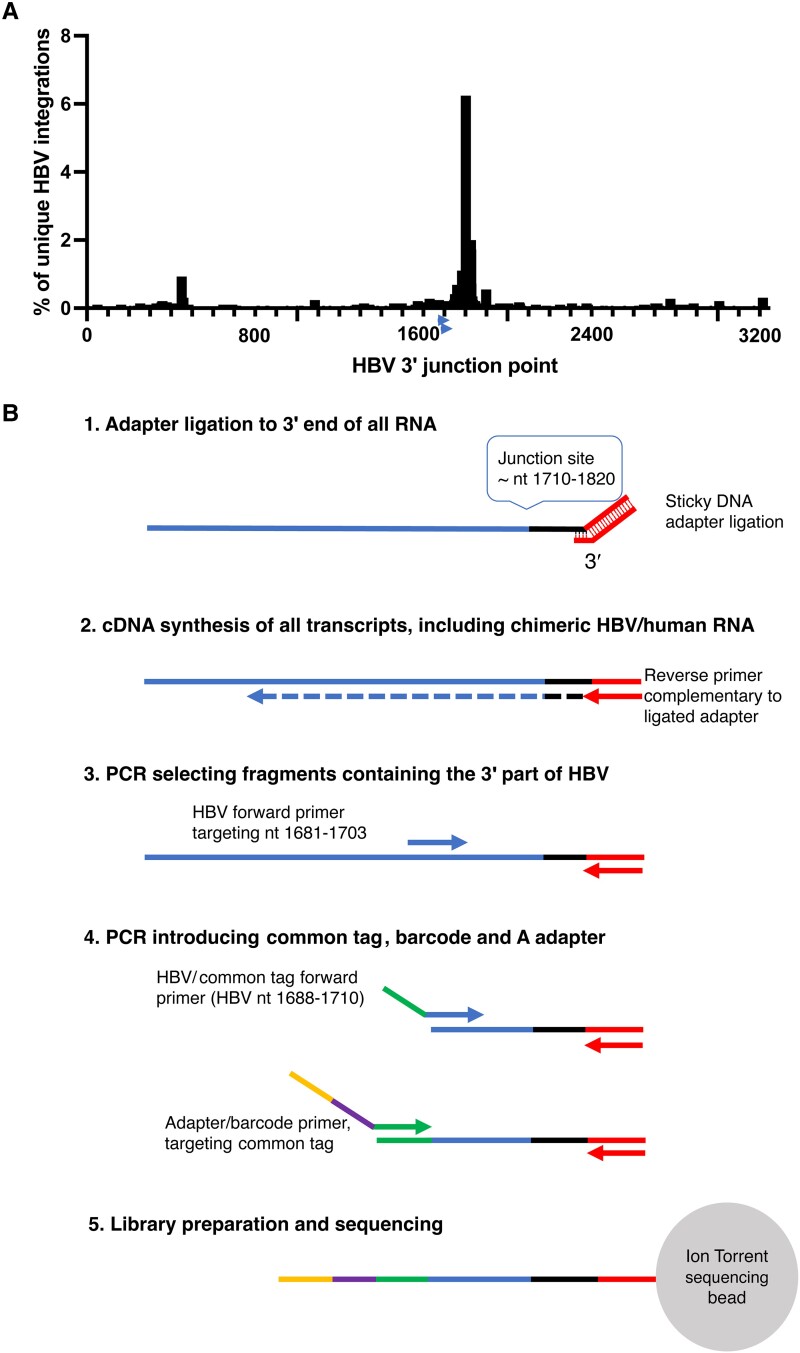
*A*, The distribution of HBV 3′ integration points (genomic position on the x-axis, percentage of all integrations on the y-axis) in chimeric HBV/human transcripts in a dataset consisting of 2899 unique HBV integrations (previously published by Ringlander et al and Furuta et al [19]). The arrows near position 1700 indicate the location of the HBV primers used in this study. *B*, HBV RNA enrichment adapted for Ion Torrent sequencing schematically depicted. HBV RNA transcribed from cccDNA as well as from HBV integrations will be amplified. The HBV-specific primers are located upstream of the region with the highest number of HBV integration points allowing detection of most of the integrations. Abbreviations: cccDNA, covalently closed circular DNA; HBV, hepatitis B virus; PCR, polymerase chain reaction.

A method for targeted identification of RNA transcribed from integrated HBV DNA by Ion Torrent sequencing (Thermo Fisher) was developed. A shown in Figure 1, the method was based on selective amplification of RNA that contains a segment (nt 1681–1710) of the X gene of HBV located upstream of the main integration site near nt 1800–1820. RNA was fragmentated using RNAase III (Thermo Fisher) and purified using the Ampure XP beads (Beckman Coulter). A double-stranded DNA oligonucleotide with the Ion Torrent P1 adapter sequence and an overhang of 6–14 random nucleotides was used to ligate the P1 adapter to the 3′ end of all RNA fragments using T4 RNA ligase (Thermo Fisher): Hybridization (of the adapter’s sticky end) at 65°C for 10 minutes and 30°C for 5 minutes was followed by incubation with ligase at 30°C for 45 minutes. Reverse transcription to cDNA was then performed using P1 as reverse primer. The cDNA was purified using Ampure XP beads (Beckman Coulter). A 2-step PCR was used for the amplification of the region and attachment of Ion Torrent A adaptor, the common tag, and barcode prior to sequencing. The first PCR step was performed using an HBV-specific forward primer (HBV1681F) in combination with P1 as reverse primer. In a subsequent seminested PCR step, an inner, HBV-specific forward primer including a common tag sequence was used (HBV1688F-CT) in combination with a P1 reverse primer. In the same reaction, primers targeting the common tag containing barcode sequences were used, which enabled pooling and sequencing of multiple samples in the same reaction. Quantification of the amplicons was performed with qPCR using the A adapter forward primer, P1 reverse primer, and a probe targeting the adapter sequence. Each sample was adjusted to 100 pM to a final volume of 50 µL. Samples were pooled and loaded onto an Ion Torrent sequencing chip with the Ion Chef (Thermo Fisher). The primers are presented in Supplementary Table 1.

### HBV RNA Enrichment With Probes and Nanopore Sequencing

Three samples with enough material and sufficient RNA integrity value (RIN >5) were analyzed with Nanopore sequencing; 2 of them (from patients 2 and 4) were also analyzed with Ion Torrent. HBV RNA enrichment prior to Nanopore sequencing was performed with a MagicBeads kit (ElementZero) containing probes covering the entire HBV genome, based on a genotype D reference. The RNA was mixed with 3 times the sample volume (150 μL) of MagIC Lysis Buffer and kept at room temperature. The washed beads were concentrated on a magnetic rack for approximately 3 minutes and the wash buffer was completely removed. Seventy microliters of the sample was added to the beads, and the tube was then centrifuged briefly and incubated for 30 minutes in a thermoshaker at 46**°**C, shaking at approximately 1000 RPM with 1 minute on/2 minutes off cycles. The beads were concentrated on a magnetic rack for 60 seconds and the supernatant was discarded. A volume of 600 μL MagIC Wash Buffer I was added to the sample and incubated under the same conditions for 10 minutes followed by a concentration of the beads for 90 seconds before the liquid was removed. The wash steps were repeated a total of 3 times. Another 600 μL of MagIC Wash Buffer II was added and the beads were resuspended by gentle pipetting and incubated another 10 minutes under the same conditions. The beads were then concentrated for 60 seconds on the magnetic rack and the supernatant removed, followed by a resuspension in 60 μL of elution buffer (100 mL Tris-HCL pH 7.5) and a 2-minute incubation at 92**°**C. The beads were again concentrated on the magnet and the RNA product was transferred to a fresh tube. Reverse transcription of RNA and Nanopore sequencing were performed with the PCR-cDNA Barcoding Kit SQK-PCB109 (Oxford Nanopore Technologies) according to manufacturer's instructions. Samples were sequenced separately at different time points on MinIon flow cells (Oxford Nanopore Technologies).

### Bioinformatics

A bioinformatics pipeline was designed to detect and quantify chimeric HBV/human reads (https://github.com/SannaAb/Viral_Integration_Pipe and Supplementary Material). Trimmed reads were mapped to HBV genotype D (GenBank, KP32260.1) and human (GenBank, hg19 (GCF_000001405.25) references using Bowtie2 aligner (version 0.7.5a) [18]. Soft-clipped reads that partly mapped towards HBV and partly to human were extracted and junction points were recorded. Reads were filtered with the trimming tool in CLC; reads quality limit was then set to 0.05 and only reads >80 nt long were included. Trimmed reads were mapped to an HBV genotype D reference and to the human hg19 reference. In 1 case (patient 3) an HBV genotype E reference was used for mapping. In the Nanopore pipeline all chimeric HBV/human reads were included regardless of number of reads. The proportions of core, preS1, preS2, and X transcripts were calculated based on the number of reads mapped to the corresponding genomic region after subtraction of the reads representing the upstream transcript.

### Statistics

Graphs depicting correlations, simple linear regression analyses, and Mann-Whitney *U* tests for comparison of RNA levels were made using Prism (version 9 or later; GraphPad).

### Ethics

This study was approved by the Regional Ethical Review Board in Gothenburg (registration number 835-17) and all patients gave informed oral and written consent.

## RESULTS

### Enrichment of HBV RNA With Ion Torrent Sequencing

The enrichment strategy to selectively amplify RNA that contained HBV nt 1681–1710 (Figure 1) was effective because 90%–95% of all Ion Torrent reads were of HBV origin. This reflected that the ratio between reads containing HBV and reads containing only human RNA was 1000-fold higher than we observed by standard Illumina RNA sequencing in a former study [8]. The mean read length of all trimmed and HBV mapped reads (including HBV/human chimeric reads) in all samples was 110 nt (range 80–384 nt).

### Extensive But Variable Expression of HBV Integrations

**Figure 2.**
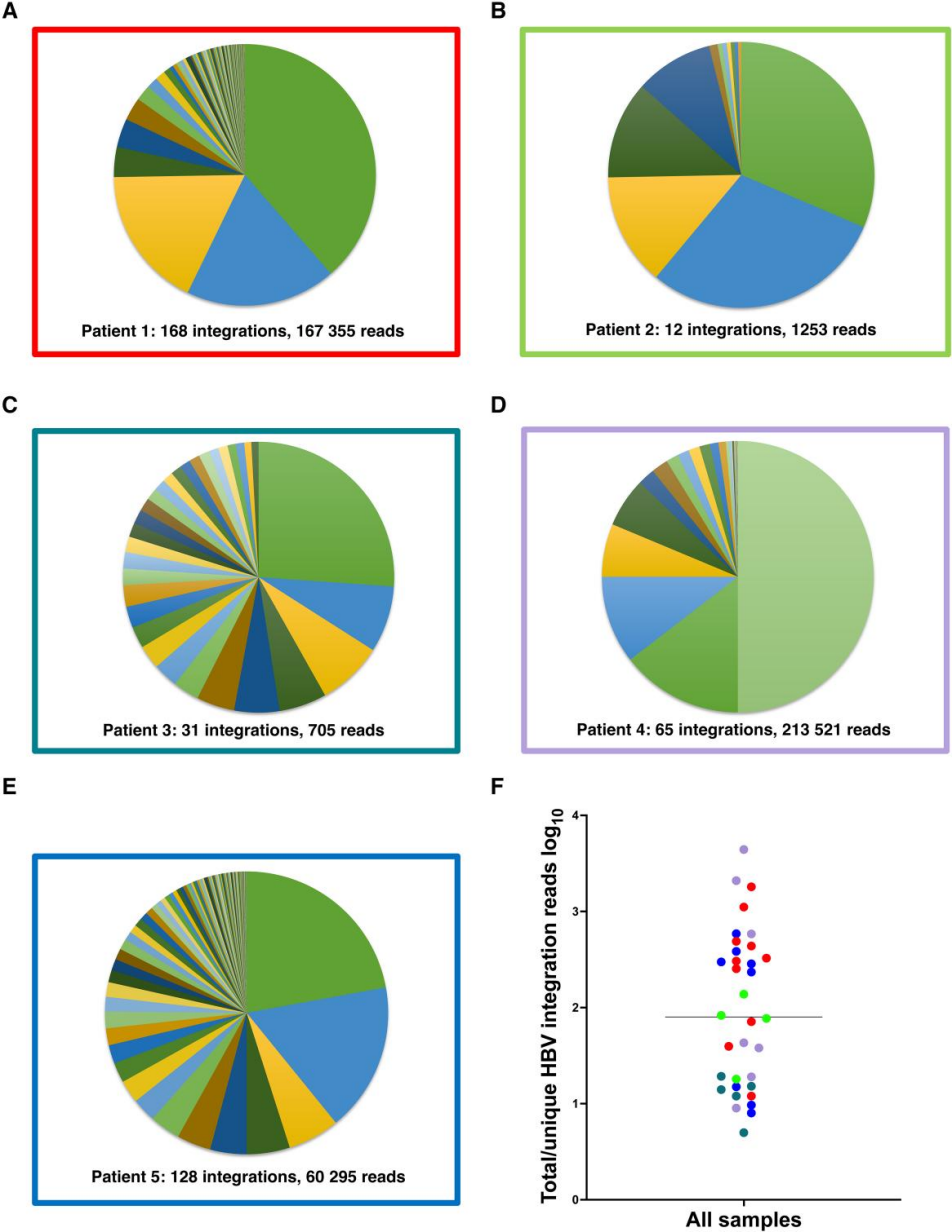
*A*–*E*, Distribution of reads between the HBV integrations identified in each patient with HBV/HDV coinfection. *F*, Ratio between the total number of integration reads and total number of unique HBV integrations for each sample, presumably reflecting the expression ratio and clonal expansion of the unique integrations. Colors represent patients 1–5 as in borders of *A*–*E*.

Reads containing an HBV sequence were detected in 45 of 50 liver tissue samples and chimeric HBV/human reads (with ≥5 coverage) were identified in a total of 35 samples. A unique HBV junction site supported by at least 5 reads was detected at a total of 404 human genome positions in tissue pieces from the 5 patients. The number of unique integrations varied between 12 and 168 per patient and the number of HBV/human chimeric reads varied between 705 and 213 521 per patient (Figure 2 and Table 2). The expression differed greatly between the integrations. While many integrations had an expression on the threshold level of 5 reads, a small minority of the integrations were the source for most of the integration-derived HBV RNA. This finding led us to expand the analysis by investigating previously published data, including patients with HBV monoinfection. RNA sequencing data from a Japanese study [19] were retrieved and analyzed, demonstrating a similar pattern with a few unique integrations contributing to most of the integration expression (Supplementary Figure 1).

**Table 2.** Deep Sequencing RNA Findings in Explant Liver Pieces From 5 Patients

Characteristics	Patient 1	Patient 2	Patient 3	Patient 4	Patient 5
HBV reads, median (range)	200 885 (249–6 124 120)	10 250 (0–601 358)	4718 (14–101 365)	19 635 (20–803 895)	124 053 (346–510 396)
Human reads, median (range)	20 443 (13–59 862)	1666 (27–98 408)	258 (10–27 928)	1151 (2–100 056)	8012 (61–51 219)
HBV reads/human reads ratio, median (range)	16 (7.6–54.5)	17.4 (0–97.0)	35 (0–70.2)	9 (3.6–25.1)	16.3 (5.7–48)
Total integration reads, median (range)	12 446 (24–61 128)	245 (0–745)	36 (0–559)	6672.5 (0–28 872)	8911.5 (0–136 816)
Unique integrations per piece, median (range)	29 (2–55)	0 (0–9)	0.5 (0–29)	12.5 (0–75)	5 (0–31)
Unique integrations in all pieces	168	12	31	65	128

### Real-Time PCR Quantification of Identified Integrations

Real-time PCR was performed on RNA and DNA from tissue samples of all 5 patients to quantify the HBV integrations that, according to the Ion Torrent RNA sequencing results, were most abundant. DNA results show the number of cells with a specific integration and RNA results the total expression of the integration. This analysis detected integration-derived RNA in 3 of 5 patients. In patient 4, one integration (in the *TAF15* gene, with a junction point at HBV nt 1797 and chr17:34157903, and with the highest number of reads [61 923] in the Ion Torrent analysis), was detected and quantified in both RNA and DNA. Approximately 1300 DNA copies of this integration in *TAF15* were detected, corresponding to approximately 1 integration copy per cell (cell count estimated by β-globin DNA real-time PCR). In this piece 560 000 RNA copy numbers of the same target were detected, indicating that there were approximately 450 RNA copies per DNA copy of this integration. DNA representing integrations in the other samples were not detected, suggesting that they were present in too few cells or that the RNA extraction might have reduced the sensitivity for DNA somewhat.

### Real-Time PCR Quantification of HBV RNA and DNA in Explant Tissue

The RNA extraction protocol that was applied for sequencing is not optimized for DNA quantification analyses. To quantify both HBV DNA and HBV RNA in liver tissue more accurately, we performed real-time PCR of the HBV S target (DNA and RNA) as well as of β-globin DNA after extraction of total nucleic acid from 10 additional tissue pieces from each of the 5 patients. The S RNA/S DNA ratio ranged between 1 and 3795 (mean 290, median 36) (Supplementary Figure 2), which agrees with the number of reads per unique integration seen in the sequencing data (mean 419, median 80, range 5–4413; Figure 2*F*) and with the integration-specific PCR result presented above. The number of HBV DNA copies per 100 cells varied greatly, from <1 to >1000.

### HBV Integrations and Serum Levels of HBsAg and HDV RNA Correlate

**Figure 3.**
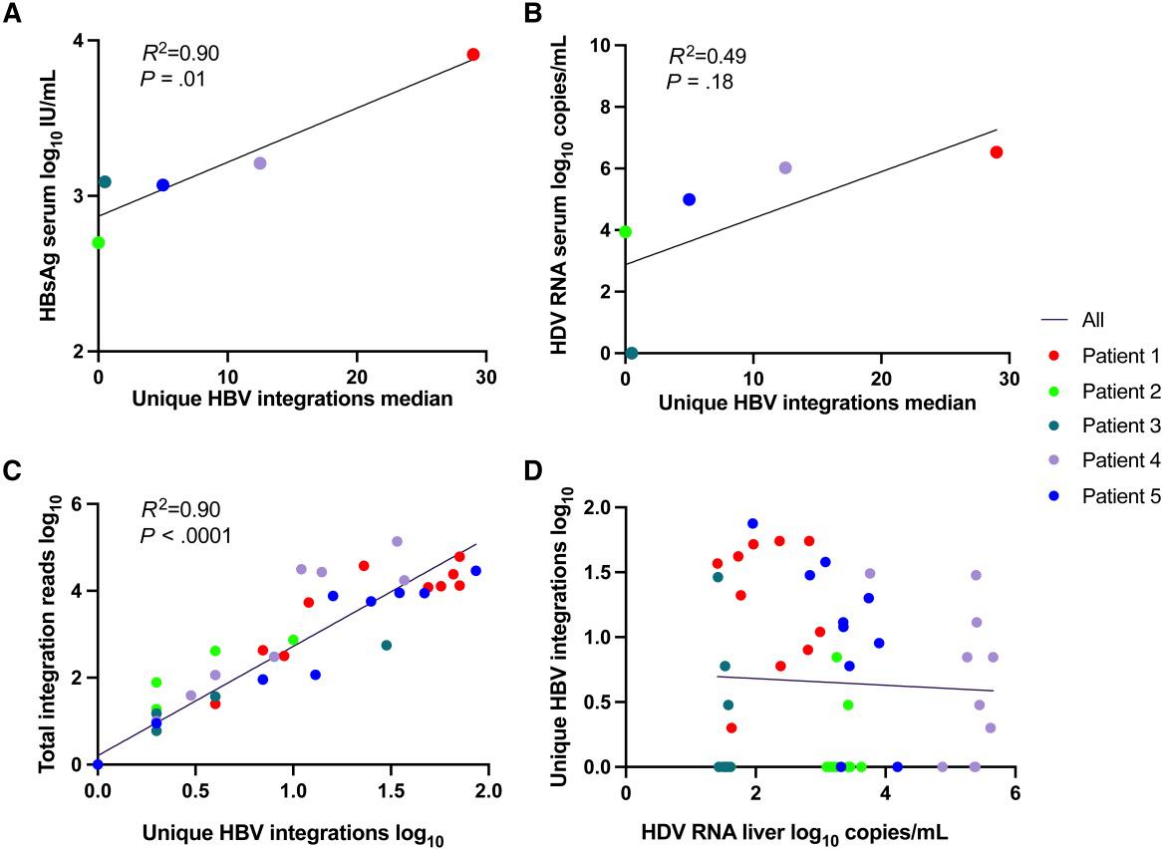
Correlation between number of unique HBV integrations in liver tissue samples and levels of (*A*) HBsAg in serum, (*B*) HDV RNA in serum, (*C*) total integrations reads, and (*D*) HDV RNA levels in liver tissue. If patient 3 who lacked HDV RNA in serum was excluded from *B*, the *R*^2^ would be 0.83 and *P* = .09. Abbreviations: HBsAg, hepatitis B surface antigen; HBV, hepatitis B virus; HDV, hepatitis D virus.

As shown in Figure 3*A* and 3*B*, the median number of unique HBV integration reads in liver explant tissue correlated with the serum level of HBsAg in samples taken at the time of the transplantation (*P* = .01) and tended to correlate with HDV RNA in serum. Within the tissue samples, the number of unique HBV integrations correlated significantly with the total number of integrations reads (Figure 3*C*) but not with the HDV RNA level (Figure 3*D*). In patient 3, who had a low number of identified HBV integrations, HDV RNA was detected in liver tissue but not in serum.

### Long-Read Sequencing of HBV RNA Shows High Ratio of Integration-Derived Transcripts

**Figure 4.**
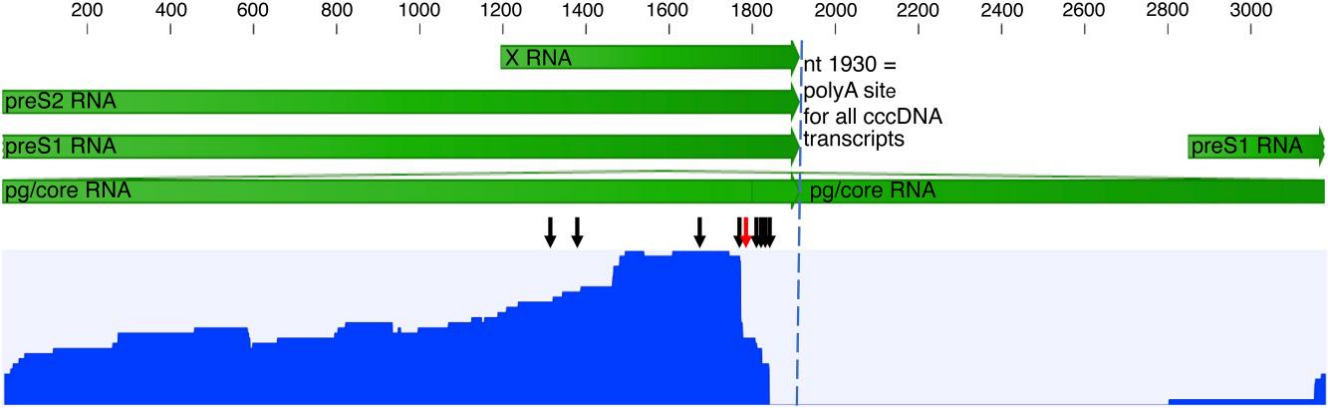
Nanopore long-read sequencing of reverse-transcribed HBV RNA (cDNA), after probe enrichment but without an initial PCR step, performed on 3 samples. The blue graph shows the reads coverage over the HBV genome for merged data from all 3 samples (rang, 0–41 reads). Most of the reads represent chimeric HBV/human transcripts starting in preS2 or X, and only 3% were transcripts starting in preS1. The black arrows indicate the identified HBV 3′ integration junction points and the red arrow a premature poly(A) site at nt 1778 observed in 1 sample. No reads included nt 1820–1930, typical for all RNA species transcribed from cccDNA (dashed vertical line), which in combination with the lack or core reads indicates that the transcripts were mainly from integrated HBV DNA. The scale and green arrows show HBV mRNA annotation. Abbreviations: cccDNA, covalently closed circular DNA; HBV, hepatitis B virus; PCR, polymerase chain reaction.

To explore the impact of HBV integrations on the HBV transcriptome profile, probe-based HBV RNA enrichment Nanopore sequencing was performed on a subset of the samples. Tissue samples from patients 2, 4, and 5 were selected (based on sufficient RNA integrity values) for HBV RNA enrichment with MagicBeads probes and Nanopore cDNA sequencing. One integration was found in each of patient 4 and 5, both of which had also been detected with Ion Torrent. An integration in the *PKIB* gene in a tissue sample from patient 4 had an HBV 3′ junction point at nt 1813, and an integration in *PDLIM5* in a sample from patient 5 had an HBV 3′ junction point at nt 1842. In patient 2 (a sample that was not Ion Torrent sequenced), 6 integrations were found (in an intergenic region of chromosome 13 with HBV 3′ junction points at nt 1344, 1495, and 1808, and in an intergenic region of chromosome 13 with the HBV 3′ junction point at nt 1768). The RNA long reads coverage profile presented in Figure 4, which includes data from all Nanopore-sequenced samples, shows higher coverage in preS2 and X (approximately 97% of all reads) than in preS1 (approximately 3% of all reads) and a lack of core/pgRNA reads. In the sample from patient 5, a premature polyadenylation site at HBV position nt 1777 was present in a minority of transcripts with an alternative poly(A) signal (AUUAAA) 15 nucleotides upstream, at nt 1757–1762.

## DISCUSSION

In this study, we developed and applied a method for Ion Torrent sequencing of HBV integration transcripts that increased the yield of integration-derived RNA by a factor of more than 1000 compared with our previous deep-sequencing analysis of similar samples [8]. Accordingly, we found an equal or greater number of unique integrations per sample and a higher total number of integration-derived reads than in previous studies of HBV integrations [8, 19–25]. The results suggest that HBV DNA integrates in a large proportion of hepatocytes, but that the HBsAg output from these cells vary >100-fold depending on clone size and expression rate. A relatively small proportion of the integrations thus seems to determine the serum levels of HBsAg and HDV RNA in coinfected patients.

The number of both unique and total integration reads differed strongly between patients and between liver tissue pieces from individual patients, and the number of reads per unique integration accordingly varied greatly. On average (median) there were 89 integration reads per unique integration, which agrees with previously reported S RNA/S DNA ratios [26], and is similar to the ratio between S RNA and S DNA (median 30) that we observed by real-time PCR quantifications of 10 additional pieces from each patient (after total nucleic acid extraction). Both number of reads/unique integration and S RNA/S DNA ratios varied greatly, from below 10 to more than 3000, suggesting that some integrations were much more expressed than other. The most abundantly expressed integration was successfully quantified regarding both RNA and DNA by a real-time PCR designed to be specific for this target, showing 450 copies of RNA per integration and on average 1 integration copy per hepatocyte. The number of HBV DNA copies per 100 hepatocytes (which is a proxy for the percentage of cells with an integration since the low levels of core RNA, shown in Figure 4, indicate the presence of very few cccDNA copies) also showed large variation (1–100), both between patients and between the different tissue pieces from individual patients. The finding suggests that many hepatocytes lack integrations whereas others may have accumulated several. Taken together, these concurring results from analyses by RNA sequencing after enrichment and by quantification of HBV S RNA and S DNA from of multiple tissue pieces show that there was large heterogeneity in the distribution and expression of integrated HBV DNA. The very high number of deep sequencing reads that was observed for some integrations (up to over 60 000 reads) was probably a result of both clonal expansion of hepatocytes with the integration and enhanced expression from these integrations [3], and suggests that a major proportion of the HBV RNA originates from just a few unique integrations.

The number of both unique integrations and total integration reads correlated with serum levels of HBsAg. Previous studies suggest that the integrations contribute to the majority of HBsAg [8, 27] and that HDV particles are composed of integration-derived HBsAg [11]. Our findings indicate that the total number of integrations and the high degree of expression of some of them determine the serum level of subviral particles with HBsAg. This indirectly suggests that the HBsAg level in serum might be viewed as a proxy for the total number of integrations. The finding that integrations correlate with the serum level of HBsAg and tend to correlate with serum HDV RNA levels indicates that the total number of integrations influences the amount of HDV that is secreted to the blood.

The Nanopore analysis showed that preS1 RNA reads constituted approximately 3% of all HBV RNA reads. PreS1 RNA is translated to L-HBsAg, which is the least abundant HBsAg in the HDV particle but necessary for HDV infection because it binds to the NTCP receptor. The low proportion of preS1 reads fits well with previous data showing that the delta virion is composed of approximately 94% S-HBsAg, 5% M-HBsAg, and 1% L-HBsAg [28]. The promoter of preS1 is present in both cccDNA and in most integrated HBV DNA, but the absence of core RNA by Nanopore suggests that the integrations were the main source of preS1-RNA and large S in the analyzed tissue. The results thus support that HDV particles may be produced also by hepatocytes without cccDNA. Similarly, we have in previous ddPCR analyses found absence or extremely low levels of core RNA in HDV-infected patients with relatively high levels of HDV RNA in serum [17]. Interestingly, there was no correlation between integration-derived reads and HDV RNA levels in liver tissue, supporting that incomplete HDV replication (multiplication of the HDV genome without secretion of viral particles) may occur even in the absence of HBsAg, as previously suggested [14, 17, 29, 30].

The Nanopore sequencing strategy produced much fewer HBV reads and a smaller number of total and unique integrations than the short-reads approach. This is probably mainly due to the lack of amplifications steps in the Nanopore protocol, a difference from the Ion Torrent method. However, the Nanopore results are important because they describe the full length of chimeric reads and revealed a small fraction of prematurely poly(A)-tailed transcripts in a sample with a high number of chimeric HBV/human reads and no or very low levels of HBV core RNA (indicating transcription from integrated HBV DNA). This observation might reflect that the poly(A) signal AUUAAA at nt 1757–1762 [31] is used more often during transcription of integrated HBV DNA than of cccDNA [32, 33]. If so, RNA sequencing could underestimate the true number of HBV integrations, because in RNA with premature polyadenylation an HBV/human junction point would not be detected.

A limitation of our study is that all patients had cirrhotic livers, which might contain a higher degree of clonal expansion of cells with integrations than noncirrhotic tissue. Another limitation is the low number of patients, and that few HBV and HDV genotypes were represented. All patients had low HBV levels in serum, probably mainly due to antiviral treatment, but interference from HDV might also have reduced the HBV DNA levels in serum and tissue [34–37].

In summary, by using a method for the enrichment of chimeric HBV/human RNA we found that a few highly expressed integrations produced most of the HBsAg in patients with liver cirrhosis, and that HBsAg and HDV RNA levels were associated with the number of integrations and their expression.

## Supplementary Data

Supplementary materials are available at *The Journal of Infectious Diseases* online (http://jid.oxfordjournals.org/). Supplementary materials consist of data provided by the author that are published to benefit the reader. The posted materials are not copyedited. The contents of all supplementary data are the sole responsibility of the authors. Questions or messages regarding errors should be addressed to the author.

## Supplementary Material

jiae045_Supplementary_Data

## Notes

***Author contributions*.** G. R., L. S., J. R., and M. L. designed the study and coherent methods. L. S., J. S., and M. A. performed sequencing and PCR analyses. S. A. and J. R. conducted bioinformatical analyses. M. L. and J. R. performed statistical analyses. C. S. and M. C. were responsible for organizing the study and recruiting patients. J. R., M. L., and S. L. performed sample collection. J. R., G. R., and M. L. wrote the manuscript draft and all authors contributed to the final version.

***Data availability*.** Sequencing data have been uploaded to the NCBI Sequence Read Archive (BioProject ID PRJNA1015140).

***Financial support*.** This work was supported by the ALF Agreement between the Swedish state and university hospitals (grant number ALF-LUA 275108 to M. L.); the Gothenburg Medical Society (to J. R.); and Cancerfonden (the Swedish Cancer Society) (grant number CAN 20 1280 PjF to M. L.). Funding to pay the Open Access publication charges for this article was provided by Swedish Universities through the Bibsam Consortium.
