# Supplementary material for: Enrichment Reveals Extensive Integration of Hepatitis B Virus DNA in Hepatitis Delta Virus-Infected Patients
Source: J Infect Dis. 2024 Jan 25;230(3):e684–93. doi: 10.1093/infdis/jiae045 (PMC11420801; doi:10.1093/infdis/jiae045)
Supplement: jiae045_Supplementary_Data [file jiae045_supplementary_data.docx]

**Supplementary material**

**Bioinformatics**

A bioinformatics pipeline was designed to detect and quantify chimeric HBV/human reads (<https://github.com/SannaAb/Viral_Integration_Pipe>). The common tag adapter sequence part (GCCAGGTTCCAGTCAC) of the reads from the Ion Torrent sequencing was trimmed using Cutadapt (version 1.9)[1]. The trimmed reads were mapped to HBV genotype D (GenBank:KP32260.1) and Human (GenBank: hg19 (GCF_000001405.25) references using Bowtie2 aligner (version 0.7.5a)[2]. Soft clipped reads that partly mapped towards HBV and partly to human were extracted and the junction points were recorded. Reads having an HBV/human junction point within 10 nt distance were compiled and counted as one unique integration. The integration points were annotated using Annovar (version 2015-06-17)[3]. Nanopore reads were mapped with the long reads adapted Minimap2 aligner (version 2.17)[4], but downstream analysis was the same as in the Ion Torrent data pipeline described above. The mapping coverage graph in Figure 4 was made using Minimap2-based long reads support tool in the CLC Genomics Workbench (Qiagen). Reads with the same HBV/human junction in several samples from different patients were thus filtered from the samples with a low coverage of the specific reads, if they had a higher reads coverage in another patient sample. With this setting, less than one percent of all reads were derived from amplicon contamination. All integrations with a coverage of five or more reads and an HBV 3’ junction point downstream of end of the first primer target (nt 1710) were included in the analyses. To further reduce the risk of overestimating integrations, we classified integrations in the same gene as representing one unique HBV integration even if they were not located to the exact same position in the human genome. This definition was used in all statistical analyses. For the HBV/human reads comparison, reads were filtered with the trimming tool in CLC; reads quality limit was then set to 0.05 and only reads >80 nt long were included. Trimmed reads were mapped to the HBV genotype D reference and to the human hg19 reference, respectively, coverage and reads metrics were compiled. In one case (patient 3) an HBV genotype E reference was used for mapping. In the Nanopore pipeline all chimeric HBV/human reads were included regardless of number of reads. The proportions of core, preS1, preS2 and X transcripts were calculated based on the number of reads mapped to the corresponding genomic region after subtraction of the reads representing the upstream transcript.

References

1. Martin M. Cutadapt removes adapter sequences from high-throughput sequencing reads. EMBnet 2011; 17:10-2.

2. Langmead B, Salzberg SL. Fast gapped-read alignment with Bowtie 2. Nat Methods 2012; 9:357-9.

3. Wang K, Li M, Hakonarson H. ANNOVAR: functional annotation of genetic variants from high-throughput sequencing data. Nucleic Acids Res 2010; 38:e164.

4. Li H. Minimap2: pairwise alignment for nucleotide sequences. Bioinformatics 2018;


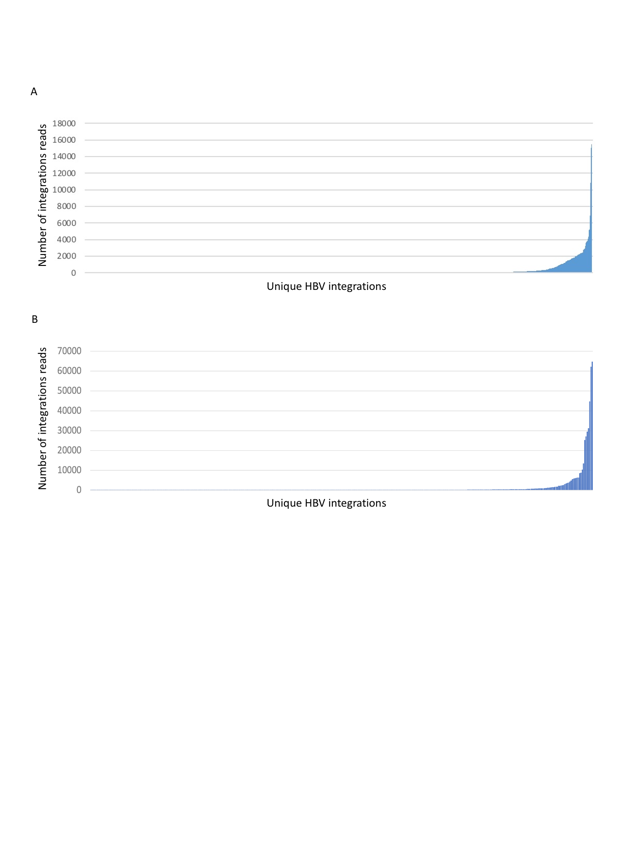


**Supplementary Figure 1**. Histogram showing the number of reads for each unique HBV integration, sorted to show integrations with the highest number of reads to the right. A) Data from Furuta *et al*. 2018. B) The present study.

**Supplementary Figure 2**. S-RNA per S-DNA in liver tissue pieces (not the same as the sequenced samples) from each of the five patients as quantified by qPCR. A) The line indicates median of all samples. B) S-RNA per S-DNA ratio in each sample C) HBV DNA (S region) per 100 cells in the samples D) S RNA per 1000 cells in the sample.

**Supplementary Table 1.** List of oligonucleotides and primers (marked F (forward) or R (reverse) used in the Ion Torrent method. The first four oligos were mixed to form a double-stranded DNA with a sticky end, which was used to ligate the P1 adapter sequence to all RNA 3’ ends. The last three oligos were used to selectively amplify cDNA fragments with both an HBV part (downstream of nt 1710) and a ligated P1 adapter sequence.

| **Oligo/primer name** | **Sequence** |
| --- | --- |
| **P1-6N** | CTCTCTATGGGCAGTCGGTGATNNNNNN |
| **P1-10N** | CTCTCTATGGGCAGTCGGTGATNNNNNNNNNN |
| **P1-14N** | CTCTCTATGGGCAGTCGGTGATNNNNNNNNNNNNNN |
| **P1 complementary** | ATCACCGACTGCCCATAGAGAG |
| **HBV1681F** | AATGTCAACGACCGACCTTGAG |
| **HBV1688-common tag F** | GACCGACCTTGAGGCATAC**CCAGGTTCCAGTCACGAC** |
| **P1 R** | CCTCTCTATGGGCAGTCGGTGAT |
